# On demand controlling of cavitation bubble collapse and jet formation through a free and rigid boundary arrangement^☆^

**DOI:** 10.1016/j.ultsonch.2025.107560

**Published:** 2025-09-19

**Authors:** Yurong Sun, Yuzhe Fan, Zhifeng Yao, Fujun Wang, Claus-Dieter Ohl

**Affiliations:** a College of Water Resources and Civil Engineering, China Agricultural University, QingHua EastRoad 17, Beijing, 100083, Beijing, China; b Faculty of Natural Sciences, Institute for Physics, Department Soft Matter, Otto von Guericke University Magdeburg, Universitätsplatz 2, Magdeburg, 39106, Saxony-Anhalt, Germany; c Research Campus STIMULATE, Otto von Guericke University Magdeburg, Otto-Hahn-Straße 2, Magdeburg, 39106, Saxony-Anhalt, Germany; d Beijing Engineering Research Center of Safety and Energy Saving Technology for Water Supply Network System beijing, Beijing, 100083, Beijing, China

**Keywords:** Cavitation bubble, Microjet, Shock wave

## Abstract

Cavitation bubbles during their collapse may form fast microscopic jet flows directed either towards a rigid boundary or away from a free surface. Here, we demonstrate experimentally that the jetting direction of a cavitation bubble near the opening of a partially liquid-filled capillary can be controlled by a non-dimensional stand-off distance, which is a function of the bubble position, capillary radius, and liquid filling. The bubble radial dynamics in the experiments are reproduced with a modified Rayleigh equation, and the full flow field is simulated with the compressible Volume-of-Fluid method. Particularly interesting cases are the neutral collapses that show either spherical symmetric flows where the partially liquid-filled capillary becomes hydrodynamically invisible to the cavitation bubble, or a torus bubble upon minimum volume, which demonstrates shock wave amplification and is similar to the one observed near a rigid boundary.

## 1 Introduction

Through the interaction between the cavitation bubble and the boundaries, bubbles produce fast and directed microscopic flows. These jet flows may be directed towards a rigid boundary [1], [2], [3] or away from a free surface [4], [5], [6], [7] and for certain geometries extreme mechanics with microjets with a speed of up to O(1000)m/s and local pressure emission in the gigapascal range have been observed [8], [9], [10], [11], [12], [13], [14]. The resulting jetting flow may be utilized for surface cleaning [15], [16] or liquid transport [2], [17].

Once the interaction of a single cavitation bubble with a simple boundary is well understood, research has increasingly shifted towards more complex geometries that are relevant to practical applications. One potential scenario involves flow confinement not by a single boundary, but by multiple boundaries. The case of two rigid walls has been studied, for example by Ref. [18], [19], [20], [21]. Once the liquid gap is below a few micrometers [22], strong fluid structure interaction has been observed. A bubble expanding nearby/in a rigid tube with/without a flow has been reported by Ref. [23], [24], [25], [26], [27], and their pumping effect in microfluidics systems has been revealed [28]. Moreover, surfaces are typically not flat but have some structure or defects. Research groups have addressed these more realistic boundaries by studying the effect of slot structures [29], [30], boundaries with through holes [17], [31], porous boundaries [32], [33], and granular boundaries [34]. When the other side of the plate is filled with gas, a violent transient liquid jet produced by flow focusing has been observed passing through the hole on the plate [35], [36], [37], [38]. When the solid boundary is coated with gas layer [39] or saturated with gas [40], the reverse of the jet direction has been revealed.

The non-ideal boundaries lead to different anisotropic pressure fields on bubble dynamics simultaneously affect strongly the energy focusing during the bubble collapse. If the boundaries are formed at a certain angle, e.g., solid corner boundary [41], [42], [43], or large-size bubble under the gravity effect near a vertical solid boundary [44], the change of the direction of the resulting liquid jets has been observed. Intriguingly, if the free and the solid boundaries are overlapped from the same direction, the resulting anisotropic pressure field can directly work on each other, and should lead to competition effect. One of the classic setup for studying such an phenomenon is the air bubble attached solid wall [45], [46], [47]. There, the cavitation bubble collapse without displacement, termed as neutral collapse, has been revealed with the stand-off parameter $\gamma =d/R_{max}$ bigger than 4.5, where d is the distance from the bubble initiation position to the proximal surface, and $R_{max}$ is the corresponding free field maximum bubble radius. At a closer distance, depending on the air bubble size, the cavitation bubble can jet towards or away from the solid surface. In the present work, we experimentally combine the anisotropic pressure field induced by a free and a rigid boundary by placing the bubble close to the opening of a partially liquid-filled capillary. Such close to a liquid-filled tube/capillary geometry, immersed into reservoir [48] or not [49], can lead to flow focusing on the concave liquid/gas meniscus [50], and has achieved the on demand droplet produce [43], [51], [52], [53] and needle free injection [54], [55], [56], [57]. There, while the transient liquid jet and its relation to the laser energy are well studied [58], [59], the influence of the complex boundaries on the bubble dynamics is largely unrevealed. Especially, the boundaries also induce a competitive anisotropic pressure field on the bubble dynamics, inducing an intriguing phenomenon, especially during the late stage of bubble collapse.

In the present study, our intent is to understand the bubble dynamics under the partially liquid-filled tube/capillary geometry. We mostly focus on the anisotropic pressure field exerted by the boundary on the bubble. While the overlapping of the anisotropic pressure field is known to change to the jet flow direction, one anisotropic pressure field always overcomes the effect of the other when they are on the same direction. In this paper, we experimentally and numerically demonstrate that, under proper geometry, the competitive anisotropic pressure fields can cancel each other, leading to neutral collapse with arbitrary stand-off distance, which, although implied by theoretical prediction [60], [61], can only be achieve in the very large stand-off distance [33]. This offers the possibility to generate strong, spherical collapses in the presence of boundaries. With further adjusting the length of the liquid plug inside the capillary, the direction and the speed of the jet can be accurately manipulated on demand. A stand-off parameter is revealed accordingly, and a modified Rayleigh equation is proposed to describe the bubble radial dynamics.

## 2 Experiment and simulation

**Fig. 1:**
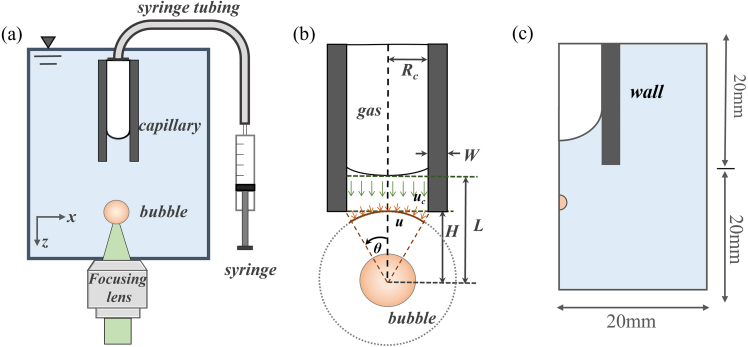
Sketch of the experimental geometry. (a) Schematic diagram of the experimental setup. (b) Schematic diagram of the studied geometry, where *H* and *L* are the distances from the bubble center to the capillary opening and the free surface. $R_{max}$ is the corresponding maximum bubble radius measured in the free field, $R_{c}$ is the inner capillary radius, and W is wall thickness. Theoretically, we assumed that the fluid velocity $u_{c}$ in the capillary is only time-dependent, and u is the radial flow velocity outside the capillary opening. θ is the angle between the line connecting the bubble center to the capillary wall and the axis of symmetry. (c) Schematic diagram of numerical domain, used 20 mm*40 mm, the angle of wedge is about 0.5 degree.

Fig. 1(a) illustrates the experimental setup: a partially liquid-filled capillary (Blaubrand, Brand GmbH) was introduced into the deionized (DI) water-filled cuvette (6 × 6 × 7 cm${}^{3}$). One end of the capillary is connected to a syringe for controlling the position of the free surface. The syringe is operated with a precision syringe pump for fine adjustment of the meniscus. A cavitation bubble is produced with a pulsed laser (Litron Nano T-250-10; λ= 532 nm; FWHM =7ns) that is focused along the axis of symmetry near the opening of the capillary with a long working distance microscope objective (5×, N.A. 0.14, Mitutoyo). The dynamics of the laser-induced bubble is recorded with a high-speed camera (Hyper Vision HPV-X2, Shimadzu) operated at a framing rate of 2 MHz with 400 × 250 pixels. Three capillaries with inner diameters $R_{c}$ of 0.27mm, 0.41mm, and 0.72mm were utilized to investigate the effect of size. Fig. 1(b) sketches the important parameters, where *H* and *L* are the distances from the bubble center to the capillary opening and the free surface, respectively. $R_{max}$ is the maximum bubble radius for the same laser energy when measured in the free field. The non-dimensional stand-off distance $\gamma =H/R_{max}$ of the bubble to the capillary opening, non-dimensional stand-off distance $\alpha =L/R_{max}$ of the bubble to the free surface, and non-dimensional inner radius of the capillary $\beta =R_{c}/R_{max}$ are conveniently normalized by the maximum bubble radius. In the present work, the parameter range of γ∈ (0∼1.3), α∈ (0∼3.2) and β∈ (0.5, 0.8, 1.4) is investigated experimentally, see Table 1.

The experimental results are compared to numerical simulations of the flow field by solving the compressible Navier–Stokes equation using the OpenFOAM framework. The solver CompressibleInterFoam is used to simulate laminar, compressible gas–liquid flow. Mass and heat transfer, as well as phase change, are neglected, while the compressibility of both phases is considered. In this model, an axisymmetric wedge of 20mm × 40mm is implemented with a minimum cell width of 1 μm, see Fig. 1(c). The bubble is seeded on the symmetry axis with an initial radius of 21.5μm. The initial pressure of the spherical bubble was set to 1.6GPa to reach a maximum bubble radius of 535μm in a free field.

**Table 1:** Values of the experimental parameters: inner capillary radius $R_{c}$, the thickness of the capillary wall W, the maximum bubble radius $R_{max}$, and the non-dimensional parameters α, β, and γ.

$R_{c}$	W	$R_{max}$	α	β	γ
0.27 mm	0.30 mm	0.53 mm	0−2.6	0.5	0−1.2
0.41 mm	0.25 mm	0.53 mm	0−3.2	0.8	0−1.4
0.72 mm	0.26 mm	0.53 mm	0−3.2	1.4	0−1.4

The corresponding equations for continuity and conservation of momentum are presented in Eqs. (1), (2).

(1) $$\frac{\partial \rho }{\partial t}+\nabla \cdot \left(\rho \vec{u}\right)=0\;$$

(2) $$\rho \frac{D\vec{u}}{Dt}=\rho \vec{f}-\nabla \vec{p}+\mu \left(\Delta \vec{u}+\frac{1}{3}\nabla \left(\nabla \cdot \vec{u}\right)\right)$$

The physical quantities involved are density ρ, time t, velocity $\vec{u}$, body force $\vec{f}$, and dynamic viscosity μ. The liquid viscosity is set to μ=1mPas and the gas viscosity to μ=0.0184mPas. For both phases, we employed Eq. (3) is as the equation of state.

(3) $$p=\left(p_{0}+B\right){\left(\frac{\rho }{\rho_{0}}\right)}^{\gamma }-B$$

For the liquid, the parameters are $p_{0}=1$  bar, B=3046  bar, $\rho_{0}=998$  kg/$m^{3}$, and γ = 7.15.

## 3 Results and discussion

**Fig. 2:**
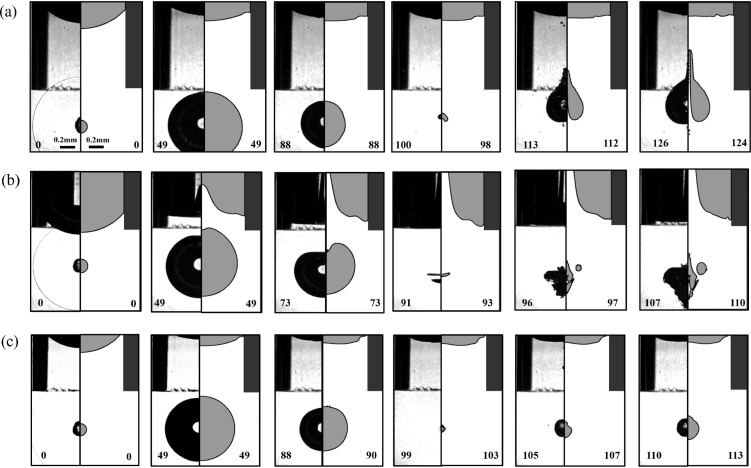
High-speed images and comparison between experiment (left) and simulation (right) revealing the jet formation for different stand-off distances α and γ. Here, $R_{max}$ = 0.53 mm, $R_{c}$ = 0.72 mm. (a) The formation of a microjet into the capillary for α∼3.1, γ∼1.0, β∼ 1.4; (b) away from the capillary for α∼1.0, γ∼1.1, β∼ 1.4; and (c) spherical bubble dynamics without jet formation for α∼2.6, γ∼1.2, β∼ 1.4. The number in each frame states the time in microseconds after the bubble initiation.

We explore the bubble dynamics as a function of the non-dimensional distance of the gas-liquid interface and the opening of the capillary, i.e., α and β. Three regimes are found: the bubble may jet into the capillary, away from it, or collapse without a center of mass translation or jet flow. Fig. 2 summarizes these three characteristic jetting dynamics by comparing selected frames from the experiments and numerical simulations before and after the first collapse in the anisotropic pressure field induced by the combined influence of the solid boundary (the inner sidewalls of the capillary) and the free surface. In the composite frames, the experiments are shown on the left and the simulations on the right. Overall, the experiments are very well reproduced by simulations, while there are some slight differences in the precise timing. We speculate that the simplification of the initial and boundary conditions in the simulations is responsible for this difference.

In Fig. 2(a), the stand-off distance from the bubble to the free surface, α∼3.1, is much larger than to the capillary opening end, γ∼1.0, causing a stronger effect of the rigid boundary compared to the free surface. The latter is displaced only by a small amount. As a result, a pronounced jet flow into the capillary is visible during re-expansion of the bubble. This geometry resembles a slot geometry [30] where jetting into the slot was observed. The case of a shorter distance to the free surface, α∼1.0, but nearly unchanged distance to the capillary γ∼1.1 is shown in Fig. 2(b). Here, due to the closer distance, the interaction between the free surface and the cavitation bubble is enhanced, causing a repulsion of the bubble from the free surface during the collapse, resulting in a jet flow away from it. Bubbles collapsing near a planar free surface form a stagnation point on the axis of symmetry along the line connecting the bubble center and the free surface. The resulting pressure gradient accelerates the bubble away from the free surface and a liquid flow towards the free surface, see e.g. Ref. [5], [62]. If the free surface has a concave shape, flow focusing leads to fast jetting from the meniscus into the air [49]. This jet into the gas phase is visible in the simulations and experiments in Fig. 2 (b), too.

At certain intermediate positions of the free surface, a force equilibrium is established between capillary-induced attraction and the opposing repulsive force from the free surface. As shown in Fig. 2(c), where α∼2.6 and γ∼1.2, there is no translation of the bubble during its collapse and rebound, and thus no jetting. Here, the bubble behaves similarly to a bubble in a free field, where the bubble maintains its spherical shape throughout the growth, collapse, and rebound phases.

**Fig. 3:**
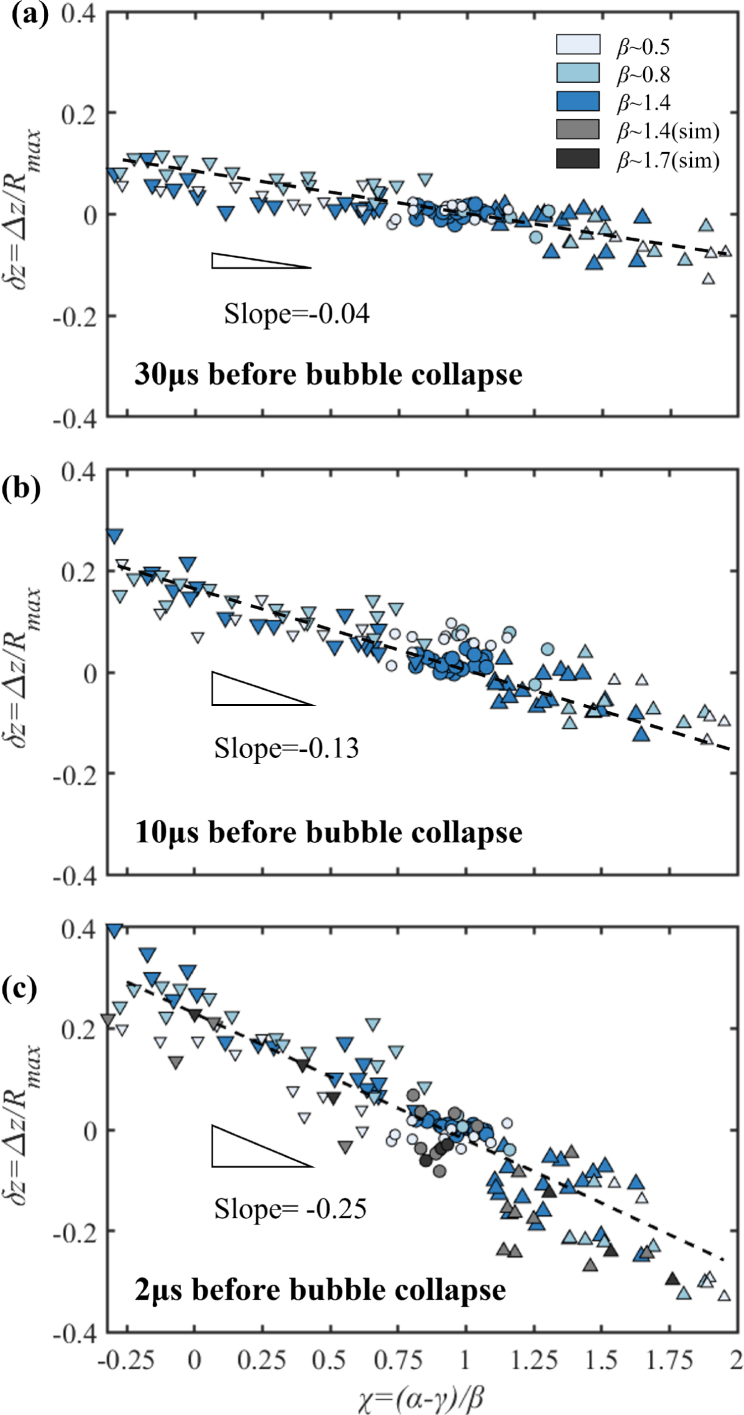
The variation of the bubble centroid displacement v.s. the proposed stand-off parameter χ at different time: (a) 30 μs before the bubble collapse; (b) 10 μs before the bubble collapse; (c) 2 μs before the bubble collapse. A downward triangle indicates that the bubble moves downward and away from the capillary; a circle indicates that the bubble collapses neutrally; an upward triangle indicates that the bubble moves towards the capillary. The dashed lines with different slopes are fitted to the data points.

Experimental and numerical results reveal that the bubble collapse and the jet formation can be determined by the difference between α and γ, which is the height of the liquid plug within the capillary. The ratio between the height of the liquid plug within the capillary and the capillary radius can be obtained from the three non-dimensional parameters introduced above, i.e., $\chi =\left(L-H\right)/R_{C}=\left(\alpha -\gamma \right)/\beta$. For small ratios of this non-dimensional liquid plug size χ, the free surface prevails, while with increasing χ, the rigid boundaries dominate. We quantify the effect of the parameter χ on the bubble dynamics through measurements of the non-dimensional displacement δz of the bubble centroid displacement Δz. Notice that, here we focus on the bubble displacement relative close to the boundaries, the cavitation bubble cannot maintain its sphericity during the second rebound in a certain range of χ, and the classical definition [32], [61] describing bubble migration is not suitable to the current situation. Here, the displacement Δz is arbitrarily chosen as the distance traveled by the bubble from its initiation position till 30μs, 10μs and 2μs before the bubble collapse. Here, a positive value describes a downward movement of the bubble during collapse and vice versa. The jetting direction is further indicated with the choice of the symbol, i.e., an upward triangle for jetting into the capillary, a downward triangle for jetting away from the capillary, and a circular symbol for the neutral collapse. The displacement δz as a function of χ is evaluated, and the results are summarized in Fig. 3. We find an approximately linear trend that an increase in non-dimensional plug size results in a movement of the bubble towards and into the capillary. A value of δz≈0 depicts no movement and thus no jetting. The neutral collapse is found in a range of 0.75≤χ≤1. The displacement of the bubble in Fig. 3 (a-c) is different at different times before bubble collapse in the non-neutral collapse regime, and leads to an increase in the absolute value of the slope of the fitted lines, and shows the development of the jet flow during the late collapse stage of cavitation bubble.

It is a surprising finding that an almost spherical collapse near a complex geometry is achievable. The effect of the partially liquid-filled capillary on the bubble dynamics can be studied with a modified Rayleigh model. Instead of spherical symmetry, we consider the kinetic and potential energy of the liquid within an opening angle of 2θ, as indicated with a orange line in Fig. 1. The angle θ is determined by the distance of the bubble center from the boundary H and the radius of the capillary $R_{C}$. Additionally, the kinetic energy of the liquid plug within the capillary with length L−H has to be taken into account. For the formula of the modified Rayleigh equation, we assume a radial velocity field outside the capillary of the $u_{r}\left(r,t\right)=R^{2}\overset{˙}{R}\;r^{-2}$, and a planar time dependent velocity field within the capillary [28], [63], $u_{c}\left(t\right)$.

Following Rayleigh [64], we apply conservation of energy, $\overset{˙}{E_{k}}+\overset{˙}{E_{p}}=0$, where $E_{p}$ is the potential energy and $E_{k}$ is the kinetic energy. The potential energy is obtained by integrating the work done to expand this section of the bubble defined by the angle θ:

(4) $$E_{p}=\Delta pV=\Delta p\int_{0}^{2\pi }\int_{0}^{\theta }\int_{0}^{R}r^{2}\sin\theta \;\text{d}r\text{d}\theta \text{d}\psi =\Delta p\left[\frac{2\pi }{3}R^{3}\left(1-\cos\theta \right)\right]$$

The kinetic energy of the liquid $E_{k}=E_{k_{1}}+E_{k_{2}}$ consists of two parts, namely, $E_{k_{1}}$ from the flow in the capillary and $E_{k_{2}}$ from the radial flow over an angle 2θ. In both volumes $V_{k_{1}}$ and $V_{k_{2}}$, we integrate the kinetic energy densities to obtain the total energies $E_{k_{1}}$ and $E_{k_{2}}$:

(5) $$E_{k_{1}}=\frac{1}{2}\rho_{l}u_{c}^{2}V_{k_{1}}=\frac{1}{2}\rho_{l}u_{c}^{2}\left[\pi R_{c}^{2}\left(L-H\right)\right]$$

(6) $$E_{k_{2}}=\int_{V_{k_{2}}}\frac{1}{2}\rho_{l}u_{r}^{2}\;\text{d}V=\int_{0}^{2\pi }\int_{0}^{\theta }\int_{0}^{\frac{H}{\cos\theta }}\frac{1}{2}\rho_{l}u_{r}^{2}r^{2}\sin\theta \;\text{d}r\text{d}\theta \text{d}\varphi =\rho_{l}\pi R^{3}{\overset{˙}{R}}^{2}\left(1-\frac{R}{2H\left(1+\cos\theta \right)}\right)\left(1-\cos\theta \right)$$

By demanding that the volume flow into the capillary is equal to the flow through the radial section, we obtain an equation for the inlet velocity into the capillary, $u_{c}$:

(7) $$\overset{˙}{R}\left[2\pi R^{2}\left(1-\cos\theta \right)\right]=u_{c}\left[\pi R_{c}^{2}\right]$$

Substituting the Eqs. (4), (5), (6), and (7) into the energy conservation law, dividing by $2\pi \rho_{l}R^{2}\overset{˙}{R}\left(1-\cos\theta \right)$ and omitting the higher-order terms in the derivative of $E_{k_{2}}$, yields a Rayleigh-type equation for the bubble radius:

(8) $$R\overset{¨}{R}\left(1+\eta -\zeta \right)+\frac{3}{2}{\overset{˙}{R}}^{2}\left(1+\frac{4}{3}\eta -\zeta \right)=-\frac{\Delta P}{\rho_{l}}\;.$$

**Fig. 4:**
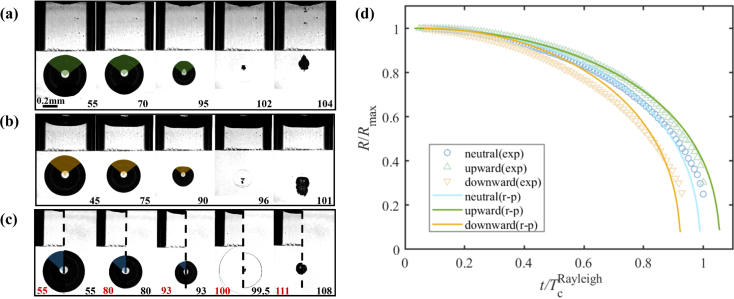
High-speed images showing the (a) jet formation towards the opening of the capillary; (b) jet formation away from the opening of the capillary; (c) spherical neutral collapse, where the comparison with composite frames of a cavitation bubble below a partially liquid-filled capillary (left) and in a free field (right) is given. The time (in microseconds) is displayed in the bottom right corner. (d) Comparison of the modified Rayleigh model, Eq. (8), and the experiments for non-dimensional bubble radius $R/R_{max}$ as a function of non-dimensional time. $T_{c}^{Rayleigh}=0.915R_{max}\sqrt{\rho_{l}/\Delta p}$ is the Rayleigh collapse time with $R_{max}=0.53\;\;\mathrm{mm}$. The radius calculated here is the equivalent radius of a part of the bubble marked with the same color in (a-c).

Here, $\zeta =\frac{R}{2H\left(1+\cos\theta \right)}$, $\eta =\frac{2R\left(L-H\right)}{R_{c}^{2}}\left(1-\cos\theta \right)=\frac{2\chi }{R_{c}}\left(1-\cos\theta \right)$, and $\Delta P=p_{\infty }-p_{V}$ is the difference between the far field pressure $p_{\infty }$ and the vapor pressure inside the bubble $p_{V}$. Please note that Eq. (8) does not account for surface tension, non-condensable gases in the bubble, viscosity, or boundary layers within the capillary. We test Eq. (8) for the radial dynamics of the three cases shown in Fig. 4 (a-c), and their radial dynamics v.s. time is given in Fig. 4(d). The cases of bubbles under spherical dynamics or mild deviations from spherical dynamics are used here. Fig. 4(d) shows for both the experiment and the modified Rayleigh equation that the downward jetting bubble collapses fastest, the upwards jetting bubble is the slowest, and the spherical collapse is in between these extremes. Notice that we also compare spherical neutral collapse with a free field collapse in Fig. 4(c). The composite images compare the shape of a bubble of the same maximum radius $R_{max}$ below the capillary (left) with a bubble in a free field (right). Both bubbles reach about the same maximum radius and collapse nearly simultaneously.

**Fig. 5:**
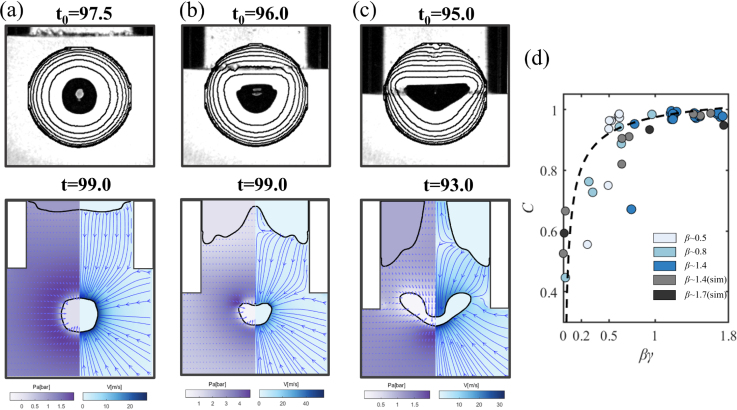
Three types of neutral collapse of the cavitation bubble, the first row is an experiment snapshot (2μs before bubble collapse), the black solid line shows the bubble contours with a time interval 6μs. The second row depicts simulation results, each frame on the left is the pressure field and on the right the velocity field. (a) α∼ 2.6, γ∼ 1.2, β∼ 1.4, (χ=1.0) (b) α∼ 1.6, γ∼ 0.5, β∼ 1.4, (χ=0.78)(c) α∼ 1.2, γ∼ 0, β∼ 1.4 (χ=0.85) and (d) the bubble shape factor C of neutral collapse v.s. βγ. Time unit: μs.

The neutral collapse occurs for 0.75≤χ≤1. When the bubble is created closer to the capillary, i.e., decreasing γ, more of the flow is confined. We further study the neutral collapse for different γ-values, see Fig. 5 (a-c). The top row of Fig. 5 (a-c) depicts three experiments, where the contours in steps of 6 μs are overlaid. Common to the three cases shown in Fig. 5 is that the bubble does not translate during the collapse. Yet they reveal different shapes once approaching minimum volume: (a) a spherical shape, (b) an inverted mushroom shape, and (c) for γ≈0 the capillary largely restricts the flow from the sides, resulting in a toroidal bubble just before collapse. Thus, through controlling the bubble dynamics, rich flow motion can also be achieved with potential applications [27], [65].

**Fig. 6:**
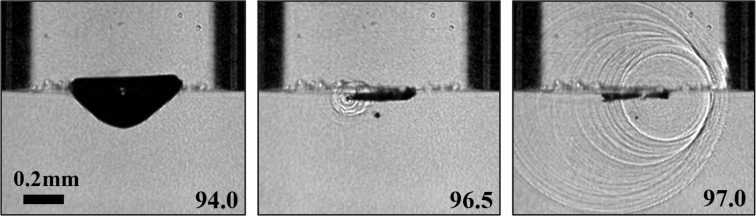
Shock wave self-focusing of a bubble under partially liquid-filled capillary, α∼1.6,γ∼0,β∼1.4.

For further analysis of the neutral collapse, we look at the roundness of the bubble using a parameter $C=4\pi A/P^{2}$, where P is the perimeter of the bubble and A is the area extracted from the high-speed recordings. As a result, when the bubble is perfectly spherical, C=1, in all other situations C<1. Fig. 5(d) plots this roundness C measured 2 μs before the neutral bubble collapse. We can express the confinement on the bubble by comparing the surface area of the bubble with the area that is below the capillary, i.e., the $R_{c}H/R_{max}^{2}=\beta \gamma$. The smaller the bubble is compared to the size of the capillary or the further it is away from the capillary, the less it will experience the radial constraint of the flow by the capillary, reducing the spherical symmetric inflow. This constraint is visible from the streamlines presented in Fig. 5(b) and (c). We therefore plot C as a function of βγ in Fig. 5(d). As βγ increases, C gradually approaches 1, hiding the hydrodynamic effects of free and rigid boundaries. On the other side, as βγ is decreasing, C gradually approaches 0, leading to a toroidal bubble prior to the collapse. The torus bubble is generally unstable [66], and asymmetries inherently present in the experiments lead to a destabilization of the torus shape. The successive collapse of the torus may lead to a shock wave self-focusing, which has been revealed near rigid boundaries [11]. Fig. 6 depicts such a self-focusing. There, the torus bubble starts to collapse at its left side (Fig. 6, t=96.5μs) where the initial emission of shock waves can be observed. These progressively and superimposedly compress the remainder of the torus, leading to the formation of an intense shock front eventually on the left side of the torus bubble in Fig. 6, t=97.0μs. The neutral, toroidal collapse allows the generation of shock wave self-focusing away from a boundary to study the pressure amplification in a liquid only, i.e., via light emission or pressure measurements [67].

Let us now discuss the limit cases not studied here. When the capillary is much smaller than the bubble, a robust pumping phenomenon has been reported [68], [69], where the liquid volume plays a less important role in the bubble dynamics [37]. Such a conclusion is also implied by our non-dimensional parameter χ, where a negligible capillary inner diameter leads to χ→∞. Then the bubble always jets towards the capillary. Additionally, modifying the wettability of the capillary has been utilized in cavitation-driven flows [49], [70]. This will affect the curvature of the liquid-air interface and thus the flow created by the pressure impulse [50].

## 4 Conclusions

In this work, we demonstrate experimentally and numerically that the translation and thus the jetting of a cavitation bubble can be achieved with mixed boundaries, i.e., a free surface and a rigid boundary as found from a partially liquid-filled capillary. The capillary diameter should be of a similar size as the cavitation bubble. Then the jetting direction is controlled by the non-dimensional parameter χ, which is the ratio of the length of the liquid plug and the radius of the capillary. For small χ, we obtain jetting away from the capillary, and for large χ into the capillary. This simple fluid mechanical system allows directing a pulsatile flow, either into the capillary or away from it, by changing the position of the bubble or the length of the liquid plug. This may offer a convenient way to utilize cavitation bubbles for suction into or jetting out of a tube.

In addition, for 0.75<χ<1 the bubble collapses without a net translation, either as a spherical bubble or in the shape of a torus. The boundaries become “invisible” when the bubble is sufficiently far from the capillary, i.e., βγ>1. Then it collapses as being present in an infinite liquid. Yet, when the bubble is closer to the capillary, it collapses as a torus. This intrinsically unstable collapse leads to a constructive superposition of shock waves, likely amplifying the pressure emitted in this regime. This resembles the collapse of an erosive bubble very close to a solid boundary. The flow field and bubble dynamics have been confirmed with volume of fluid simulations. Additionally, we presented a Rayleigh-type model demonstrating the reduction or increase of the lifetime of the bubbles as a function of jetting direction.

We may speculate that the neutral collapse could have implications for designing surfaces that mitigate cavitation erosion. In particular, a bubble that does not jet also does not migrate to the wall. An earlier study [39] by some of us reported that a combination of a rigid and a free boundary may lead to the repulsion of the bubble. There, the rigid boundary is parallel to the free boundary, while in the present study, the rigid boundary is normal to the free boundary. If one replicates the geometry, we could have a porous boundary that is much simpler to manufacture than the complex biomimetic surface in Ref. [39].

## CRediT authorship contribution statement

**Yurong Sun:** Writing – original draft, Software, Investigation. **Yuzhe Fan:** Writing – review & editing, Supervision, Project administration. **Zhifeng Yao:** Project administration, Funding acquisition. **Fujun Wang:** Project administration. **Claus-Dieter Ohl:** Writing – review & editing, Resources, Conceptualization.

## Declaration of competing interest

The authors declare that they have no known competing financial interests or personal relationships that could have appeared to influence the work reported in this paper.
